# Minimal Change Disease With Mesangial IgA Deposition: A Clinicopathological Case Series From a Tunisian Referral Center

**DOI:** 10.1155/crin/7021133

**Published:** 2026-09-22

**Authors:** Meriam Hajji, Ilhem Ben Othman, Abir Boussetta, Rihab Zouaoui, Nada Sellami, Hayet Kaaroud, Taher Gargueh, Ezzeddine Abderrahim

**Affiliations:** ^1^ Department of Nephrology and Internal Medicine, Charles Nicolle Hospital, Tunis, Tunisia, chucharlesnicolle.tn; ^2^ Kidney Pathology Laboratory LR00SP01, Charles Nicolle Hospital, Tunis, Tunisia, chucharlesnicolle.tn; ^3^ Faculty of Medicine of Tunis, El Manar University, Tunis, Tunisia, utm.rnu.tn; ^4^ Department of Paediatrics, Charles Nicolle Hospital, Tunis, Tunisia, chucharlesnicolle.tn

**Keywords:** IgA nephropathy, immunofluorescence, kidney biopsy, mesangial IgA deposition, minimal change disease, nephrotic syndrome

## Abstract

**Background:**

Minimal change disease (MCD) associated with mesangial immunoglobulin A (IgA) deposition, historically referred to as “IgA Nephrosis,” is a rare and nosologically controversial entity. Whether it represents MCD with incidental mesangial IgA deposition, a dual glomerulopathy combining MCD and subclinical IgA nephropathy (IgAN), or a distinct clinicopathological phenotype remains uncertain. Only isolated case reports and small series have been published to date.

**Methods:**

We retrospectively identified six consecutive patients (three children/adolescents and three adults; four males and two females) evaluated at the Kidney Pathology Laboratory of the Department of Nephrology, Charles Nicolle Hospital, Tunis, between 1995 and 2025. All patients presented with nephrotic syndrome, minimal or absent glomerular abnormalities on light microscopy, and dominant mesangial IgA deposits on direct immunofluorescence.

**Results:**

Microscopic hematuria was present in three patients, while one patient reported a remote episode of macroscopic hematuria during childhood. Light microscopy revealed essentially normal glomeruli in three cases and only mild mesangial hypercellularity and/or matrix expansion in the remaining cases, without endocapillary proliferation, crescents, necrotizing lesions, or significant chronic damage. Immunofluorescence consistently demonstrated dominant or exclusive mesangial IgA deposits, variably associated with IgM, IgG, C3, C1q, fibrinogen, or light‐chain co‐deposition. Electron microscopy was unavailable. Most patients exhibited steroid‐sensitive disease, although relapsing courses were frequent.

**Conclusions:**

MCD with mesangial IgA deposition represents an uncommon clinicopathological entity situated at the interface between MCD and IgAN. The marked discrepancy between severe nephrotic syndrome and minimal histological lesions despite mesangial IgA deposition strongly suggests a predominant podocytopathy rather than classical proliferative IgAN. Recognition of this phenotype is important to avoid diagnostic misclassification and inappropriate therapeutic strategies. Further studies integrating ultrastructural and molecular analyses are required to clarify its pathogenesis and nosological position.

## 1. Introduction

Minimal change disease (MCD) and immunoglobulin A (IgA) nephropathy (IgAN) are among the most common primary glomerular diseases worldwide, yet their co‐existence within the same kidney biopsy remains uncommon and pathophysiologically intriguing. MCD is classically characterized by nephrotic syndrome associated with normal or near‐normal glomeruli on light microscopy, absence of significant immune deposits on immunofluorescence, and diffuse podocyte foot‐process effacement on electron microscopy (EM). In contrast, IgAN is defined by dominant or co‐dominant mesangial IgA deposits, frequently accompanied by C3, IgG, IgM, or light chains, with variable proliferative lesions on light microscopy ranging from mild mesangial hypercellularity to crescentic glomerulonephritis.

The occurrence of nephrotic syndrome with minimal glomerular abnormalities despite dominant mesangial IgA deposition therefore represents a diagnostic and nosological challenge. This clinicopathological association, historically termed “IgA Nephrosis,” has also been described as “MCD with IgA deposits,” “MCD–IgA overlap,” or “dual glomerulopathy” in the literature. Since its initial descriptions in the 1980s [1, 2], only scattered case reports and small retrospective series have been published, leaving its prevalence, pathogenesis, prognosis, and optimal therapeutic management poorly understood [3–5].

The principal challenge lies in determining whether mesangial IgA deposition in this context represents incidental nonpathogenic trapping in otherwise typical MCD, latent subclinical IgAN associated with superimposed podocytopathy, or a distinct immunopathological entity involving shared mechanisms of podocyte injury and mucosal immune dysregulation. This distinction has important clinical implications because classical IgAN with nephrotic syndrome generally demonstrates less steroid responsiveness than MCD‐associated podocytopathy [4, 5].

In this study, we report six cases of MCD with mesangial IgA deposition identified over a 30‐year period at a tertiary nephrology referral center in Tunisia. We describe their clinical, biological, and histopathological features and discuss the differential diagnosis and clinicopathological significance of this uncommon entity in light of the existing literature.

## 2. Patients and Methods

This retrospective single‐center case series was conducted at the Department of Nephrology and Internal Medicine of Charles Nicolle Hospital, Tunis, Tunisia. All native kidney biopsies performed between January 1995 and December 2025 were screened for cases fulfilling the following inclusion criteria: (1) Clinical presentation consistent with nephrotic syndrome, defined by proteinuria > 3.5 g/24 h (or urine protein‐to‐creatinine ratio > 3500 mg/g), hypoalbuminemia, and edema; (2) dominant or exclusive mesangial IgA deposits on direct immunofluorescence; and (3) absence of significant proliferative, necrotizing, or sclerosing lesions on light microscopy suggestive of classical IgAN with nephrotic‐range proteinuria according to established Oxford histopathological criteria [6].

Patients with secondary causes of mesangial IgA deposition, including IgA vasculitis and chronic liver disease, were excluded. Cases with focal segmental glomerulosclerosis beyond isolated global sclerosis were also excluded. One patient had associated celiac disease but lacked clinical, biological, or histopathological features supporting secondary IgAN.

Kidney biopsy specimens were processed according to standard histopathological protocols. Light microscopy was performed on paraffin‐embedded sections stained with hematoxylin and eosin, periodic acid–Schiff, Masson’s trichrome, and Jones methenamine silver stains. Immunofluorescence studies were carried out on frozen sections using fluorescein‐labeled polyclonal antibodies directed against IgA, IgG, IgM, C3, C1q, fibrinogen, and kappa and lambda light chains. Staining intensity was graded semiquantitatively from 0 to 3+ according to standard nephropathology practice [7]. EM was unavailable throughout the study period.

Clinical and laboratory data, including demographic characteristics, clinical presentation, biochemical parameters, treatment modalities, and renal outcomes, were retrospectively collected from medical records. The study was approved by the institutional ethics committee, and all patient identifiers were anonymized before analysis.

The study was conducted in accordance with the Declaration of Helsinki and approved by the Institutional Ethics Committee of Charles Nicolle Hospital, Tunis. Owing to the retrospective nature of the study, the use of anonymized data, and the inclusion of patients over a prolonged study period (1995–2025), obtaining informed consent from all participants was not feasible.

## 3. Case Reports

Six patients fulfilling a clinicopathological phenotype consistent with MCD associated with mesangial IgA deposition were identified. The cohort included four males and two females, with ages ranging from 10 to 36 years at the time of kidney biopsy. All patients presented with nephrotic syndrome, including nephrotic syndrome with proteinuria associated with hypertension, hematuria, or acute kidney injury in four cases. Microscopic or macroscopic hematuria was documented in four patients, hypertension in two, and acute kidney injury in one. Nephrotic‐range proteinuria was present in all cases, whereas renal function was preserved in the majority of patients (Table 1).

**TABLE 1:** Clinical, biological, and histopathological characteristics of patients with minimal change disease associated with mesangial IgA deposition.

Variable	Case 1	Case 2	Case 3	Case 4	Case 5	Case 6
Age at biopsy (years)	10	15	16	27	36	26
Sex	Male	Male	Male	Male	Female	Female
Age at disease onset (years)	4	1	15	21	36	Childhood (macroscopic hematuria)
Clinical presentation	Relapsing NS	Relapsing NS	NS + HTN	Relapsing NS	Anasarca	Impure NS
Number of relapses	7	Multiple	1 previous episode	Multiple	None	Relapse after MMF withdrawal
Hypertension	Yes	No	Yes	No	No	No
Hematuria	Microscopic	Absent	Absent	Microscopic	Microscopic	Microscopic + macroscopic hematuria
24‐h Proteinuria (g/day)	3	3	6	Not available	9	3.5 (at relapse)
Serum albumin (g/L)	Not available	12	26	21	12	23 (at relapse)
Serum creatinine (μmol/L)	153	50	88	84	59	Normal
Acute kidney injury	Yes	No	No	No	No	No
Light microscopy findings	Normal glomeruli	Essentially normal glomeruli; 1 globally sclerosed glomerulus	Mild diffuse mesangial matrix expansion	Focal mild mesangial hypercellularity and matrix expansion	Normal glomeruli	Focal mild mesangial hypercellularity and matrix expansion
Endocapillary proliferation/crescents	Absent	Absent	Absent	Absent	Absent	Absent
Chronic lesions	None	Isolated global sclerosis	None	None	None	None
Predominant IF findings	IgA (2–3+); IgM (1+)	IgA (2+); IgM + κ/λ	IgA (2–3+); C3 + fibrinogen + λ	IgA (2+); IgM + C1q + κ/λ	IgA > IgG; C3+; C1q (trace)	Exclusive IgA (2–3+)
Treatment	Prednisolone + cyclophosphamide	Prednisolone	Prednisolone	Prednisolone + MMF	Corticosteroids + MMF	Corticosteroids + MMF
Clinical outcome	Steroid‐dependent relapsing course	Recurrent relapses	Favorable initial response	Partial improvement	Sustained remission	Relapse after MMF discontinuation

*Note:* Hypertension in MCD is uncommon, suggesting either coincidental essential hypertension or a more severe underlying renal pathology, though histology argued against proliferative disease.

Abbreviations: HTN, hypertension; IF, immunofluorescence; MMF, mycophenolate mofetil; NS, nephrotic syndrome; κ/λ, kappa/lambda light chains.

### 3.1. Case 1

A 10‐year‐old boy with a history of allergic rhinitis was referred for a seventh relapse of steroid‐sensitive nephrotic syndrome initially diagnosed at the age of four years. The current episode was complicated by hypertensive crisis and acute kidney injury, with serum creatinine rising to 153 μmol/L. Immunological investigations including ANA, serum complement fractions (C3 and C4), and antistreptolysin O titers were within normal limits. Twenty‐four‐hour urinary protein excretion was 3 g/day.

**FIGURE 1:**
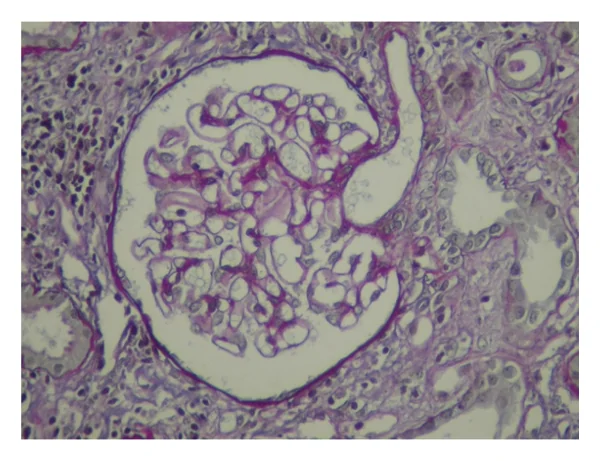
Light microscopy of a renal biopsy specimen from Case 1: periodic acid–Schiff stain (PAS, × 400); all glomeruli are morphologically normal. Mesangial cellularity and matrix content are within normal limits.

Kidney biopsy contained 15 glomeruli. Light microscopy showed no significant glomerular abnormalities (Figure 1). Tubulointerstitial and vascular compartments were preserved. Immunofluorescence demonstrated diffuse granular mesangial IgA deposits of moderate‐to‐strong intensity (2‐3+) in all glomeruli, associated with weak IgM staining (1+). Staining for IgG, C3, fibrinogen, and light chains was negative.

Overall findings supported the diagnosis of MCD associated with mesangial IgA deposition. The patient received high‐dose prednisolone followed by cyclophosphamide because of repeated disease activity during follow‐up. Despite an initial response to corticosteroid therapy, subsequent disease activity required additional immunosuppressive treatment.

### 3.2. Case 2

A 15‐year‐old male with nephrotic syndrome diagnosed during infancy presented with edema after a prolonged relapsing but steroid‐sensitive course. Laboratory investigations revealed a serum protein concentration of 43 g/L, serum albumin of 12 g/L, serum creatinine of 50 μmol/L, and 24‐h proteinuria of 3 g/day. ANA testing was negative, and complement fractions (C3, C4, and CH50) were within normal limits.

**FIGURE 2:**
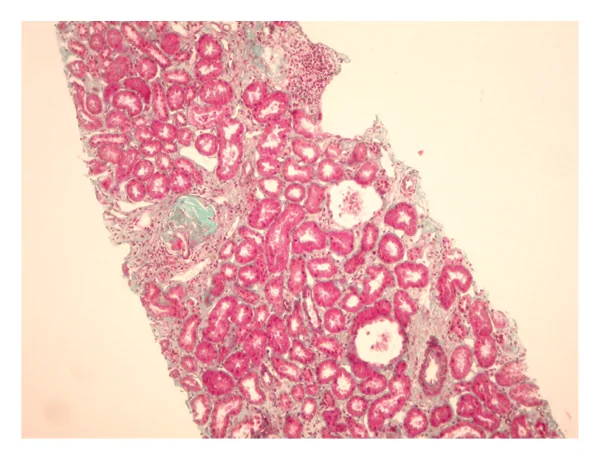
Optical microscopy reveals normal glomeruli; one glomerulus shows global sclerosis; Masson’s trichrome (× 100).

Kidney biopsy included 24 glomeruli, one of which was globally sclerosed (4.2%). The remaining glomeruli were unremarkable on light microscopy, with preserved mesangial cellularity and matrix content (Figure 2). No tubulointerstitial fibrosis or vascular abnormalities were identified. Immunofluorescence demonstrated diffuse dominant mesangial IgA deposits (2+) in all viable glomeruli, associated with mild IgM and polytypic light‐chain staining (1+ each for kappa and lambda). IgG, C3, C1q, and fibrinogen were negative.

The overall clinicopathological presentation favored MCD with mesangial IgA deposition. Full‐dose corticosteroid therapy was initiated, leading to remission; however, disease activity recurred during subsequent follow‐up. At the last follow‐up visit, serum creatinine was 55 μmol/L, and the urine protein‐to‐creatinine ratio was 0.8 g/g, indicating persistent but improved proteinuria.

### 3.3. Case 3

A 16‐year‐old male presented with nephrotic syndrome associated with hypertension (140/90 mmHg), occurring five months after a previous steroid‐responsive episode. Urinalysis revealed nephrotic‐range proteinuria without hematuria. Twenty‐four‐hour urinary protein excretion reached 6 g/day. Serum total proteins, albumin, and creatinine were 60, 26 g/L, and 88 μmol/L, respectively.

**FIGURE 3:**
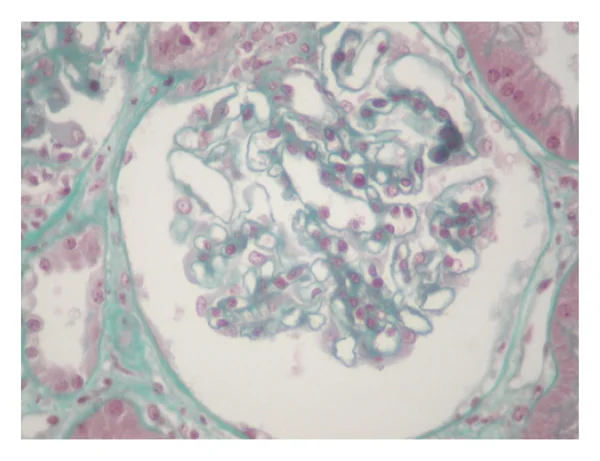
Masson’s trichrome (× 400); mild diffuse mesangial matrix expansion without mesangial hypercellularity.

Kidney biopsy contained 26 glomeruli. Light microscopy demonstrated mild diffuse mesangial matrix expansion without mesangial hypercellularity (Figure 3). Glomerular capillary loops and endothelial structures were preserved. Immunofluorescence showed dominant granular mesangial IgA deposits (2‐3+) associated with weak C3, fibrinogen, and lambda light‐chain staining. IgG, IgM, C1q, and kappa light chains were negative.

Given the marked discrepancy between severe nephrotic syndrome and minimal histological lesions, the findings were considered compatible with MCD associated with mesangial IgA deposition. Corticosteroid therapy at 1 mg/kg/day was initiated, resulting in favorable initial clinical response. Follow‐up revealed the following: Serum creatinine of 90 μmol/L and urine protein‐to‐creatinine ratio of 1.2 g/g at 3 months.

### 3.4. Case 4

A 27‐year‐old male with a history of relapsing nephrotic syndrome previously treated with corticosteroids and mycophenolate mofetil presented with edema. Blood pressure was normal, and no extra‐renal manifestations were identified. Urinalysis revealed proteinuria and microscopic hematuria. Serum total protein, albumin, and creatinine levels were 54, 21 g/L, and 84 μmol/L, respectively.

**FIGURE 4:**
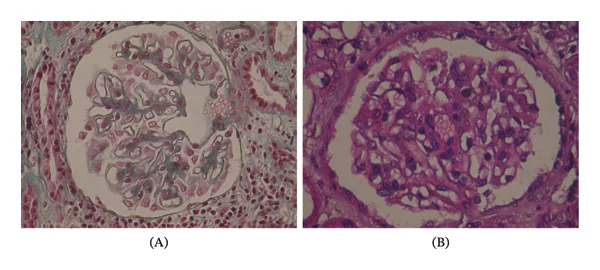
Light microscopy of a renal biopsy specimen from Case 4. (A) Masson’s trichrome (× 400). (B) Hematoxylin and eosin (× 400). Focal and segmental mesangial hypercellularity with expansion of the mesangial matrix. Mesangial cell count exceeds three cells per mesangial area in the involved segment.

**FIGURE 5:**
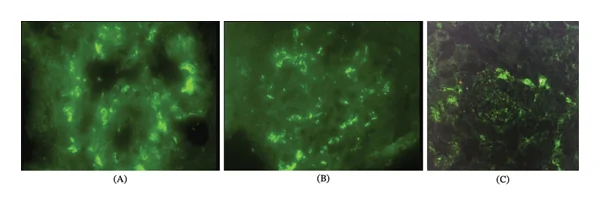
Direct immunofluorescence of a renal biopsy cryostat section from Case 4. (A) IgA (× 400): dominant granular mesangial deposits (2+) with a characteristic starburst mesangial distribution. (B) IgM (× 400): minor co‐deposits (1+) of markedly lesser intensity compared to IgA, also confined to the mesangium. (C) C1q (× 400): trace mesangial positivity (±).

Kidney biopsy yielded nine glomeruli. Two glomeruli (22%) demonstrated focal mild mesangial hypercellularity associated with mesangial matrix expansion (Figure 4). Immunofluorescence demonstrated mesangial granular IgA deposits (2+) associated with weaker IgM, C1q, and polytypic light‐chain staining (Figure 5).

The limited mesangial lesions observed on light microscopy were considered insufficient to explain the nephrotic presentation, supporting a diagnosis of MCD associated with mesangial IgA deposition. MMF was continued given prior steroid‐dependence and to minimize corticosteroid exposure, with partial clinical improvement achieved. Follow‐up revealed the following: Serum creatinine of 88 μmol/L and urine protein‐to‐creatinine ratio of 2.1 g/g at last visit.

### 3.5. Case 5

A 36‐year‐old woman with a history of urinary lithiasis and reactive cervical lymphadenopathy presented with anasarca. Blood pressure was normal. Urinalysis demonstrated nephrotic‐range proteinuria associated with microscopic hematuria. Twenty‐four‐hour proteinuria reached 9 g/day. Serum total protein was 46 g/L, serum albumin 12 g/L, serum cholesterol 10 mmol/L, and serum creatinine 59 μmol/L.

**FIGURE 6:**
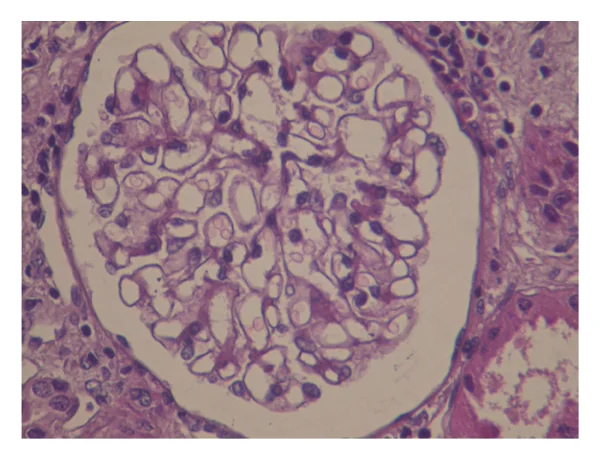
No mesangial hypercellularity, matrix expansion, or chronic sclerotic lesions. Periodic acid–Schiff stain (PAS, × 400).

Kidney biopsy included 20 glomeruli, all of which were unremarkable on light microscopy. No mesangial hypercellularity, matrix expansion, or chronic sclerotic lesions were identified (Figure 6). The tubulointerstitial and vascular compartments were preserved. Immunofluorescence revealed co‐dominant mesangial deposits, with IgA (2+) slightly predominating over IgG (1+), associated with weak C3 and trace C1q staining.

The absence of significant proliferative lesions despite massive nephrotic syndrome supported a diagnosis of MCD with mesangial IgA deposition. Following symptomatic treatment with diuretics and spironolactone, corticosteroid therapy was initiated, resulting in complete clinical and biochemical remission. No relapse occurred during 5 years of follow‐up. At the last follow‐up, serum creatinine was 62 μmol/L, and the urine protein‐to‐creatinine ratio was < 0.3 g/g.

### 3.6. Case 6

A 26‐year‐old woman presented with anasarca in the setting of nephrotic syndrome with proteinuria and microscopic hematuria. She reported a history of celiac disease and a remote episode of unexplained gross hematuria during childhood. There was no familial history of kidney disease. Blood pressure and renal function were normal, and no extra‐renal manifestations were identified.

**FIGURE 7:**
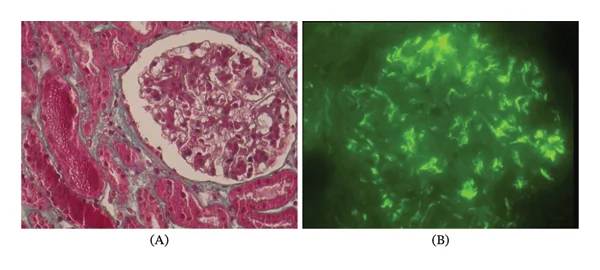
Case 6. (A) Light microscopy showing focal segmental mild mesangial hypercellularity with mesangial matrix expansion and thickening, along with mesangial deposits. A red blood cell cast is also observed adjacent to the glomerulus (Masson’s trichrome × 400). (B) Direct immunofluorescence demonstrates strong (2‐3+) granular mesangial IgA deposits in all glomeruli, including those appearing normal on light microscopy.

Kidney biopsy contained 26 glomeruli. Twenty‐three glomeruli were unremarkable, whereas three glomeruli (11.5%) exhibited focal mild mesangial hypercellularity associated with moderate mesangial matrix expansion and faint PAS‐positive mesangial deposits. Immunofluorescence demonstrated strong diffuse granular mesangial IgA deposits (2–3+) without associated IgG, IgM, C3, C1q, fibrinogen, or light‐chain staining (Figure 7).

The clinicopathological presentation supported the diagnosis of MCD associated with isolated mesangial IgA deposition. Treatment consisted of high‐dose corticosteroids followed by gradual tapering combined with MMF (2 g/day), leading to remission. Follow‐up at discontinuation revealed the following: Serum creatinine of 58 μmol/L and urine protein‐to‐creatinine ratio of 0.2 g/g. Four years later, a relapse occurred after MMF discontinuation, with recurrence of nephrotic syndrome characterized by a serum protein concentration of 49 g/L, serum albumin of 23 g/L, and proteinuria of 3.5 g/day. Re‐treatment with corticosteroids and reinitiation of MMF achieved remission.

## 4. Discussion

The combination of nephrotic syndrome, MCD‐pattern light microscopy, and dominant mesangial IgA deposits was first formally characterized in isolated case reports in the early 1980s [1] and then systematically reviewed by Clive, who collated 46 published cases from 12 countries [2]. The median age in that review was 14 years, with a male predominance (male:female ratio approximately 3:1)—a gender distribution mirrored in our series (4M:2F). Subsequent series from Korea (Cho et al., *n* = 46) [3] and single‐center East Asian reports [8] confirmed that this entity is not geographically restricted, though its true prevalence remains unknown owing to the absence of systematic registry‐based screening.

In our cohort, the age at biopsy ranged from 10 to 36 years, with presentations at various stages of relapsing nephrotic syndrome—from the first episode (Cases 5 and 6) to the seventh relapse (Case 1). The recurrent, steroid‐sensitive pattern in Cases 1 and 2 strongly resembles classical MCD behavior, whereas the microscopic hematuria in Cases 1, 4, and 5 and the remote macroscopic hematuria in Case 6 represent a clinical signature more in keeping with IgAN. These overlapping features underscore the nosological ambiguity of this syndrome.

Light microscopy in MCD with IgA deposits is, by definition, characterized by an absence or near‐absence of significant glomerular pathology. In our series, three cases showed entirely normal glomeruli (Cases 1, 2, and 5), while the remaining cases exhibited focal segmental mesangial changes—mild hypercellularity, moderate matrix expansion, or rare PAS‐positive deposits—affecting between 11.5% (Case 6, 3/26 glomeruli) and 22% (Case 4, 2/9 glomeruli) of sampled glomeruli. This focal and segmental distribution is of paramount diagnostic importance: It represents a minor mesangial signature that falls well short of the diffuse or global mesangial proliferation characterizing full‐blown IgAN (Oxford MEST: M1 lesion defined as mesangial hypercellularity in > 50% of glomeruli) [6]. The subtle LM alterations in our patients likely reflect the physical presence of mesangial immune aggregates causing mild architectural distortion, rather than a primary proliferative glomerulopathy.

Key LM features to report in suspected MCD with IgA deposits include the following: Number of glomeruli sampled (adequacy); proportion with any mesangial cell increase (Oxford MEST M‐score); absence of endocapillary proliferation (E0), segmental glomerulosclerosis (S0), tubular atrophy/interstitial fibrosis (T0), and crescents (C0)—i.e., a favorable MEST‐C profile; qualitative assessment of mesangial matrix on PAS and silver stains; and presence or absence of global sclerosis, which may reflect prior cumulative damage from relapsing disease (as in Case 2, where 4.2% of global sclerosis was present after 16 years of relapsing nephrotic syndrome).

Immunofluorescence is the cornerstone diagnostic modality in MCD with IgA deposits. By definition, IgA must be dominant or exclusive in the mesangium. In all six of our cases, IgA was the dominant or sole fluorescent reactant. Three IF patterns emerged as follows:

1. Exclusive IgA (Case 6): A strong (2‐3+) granular mesangial IgA deposition pattern was observed, with no detectable IgG, IgM, C3, C1q, fibrinogen, or light‐chain deposits. This immunofluorescence profile, characterized by isolated mesangial IgA deposition, is most consistent with an IgA‐mediated glomerular process and supports the diagnosis of IgAN in the appropriate clinical and histopathological context. The absence of light‐chain restriction does not support a monoclonal IgA deposition disorder.
2. Dominant with minor co‐deposits (Cases 1, 2, 3, and 4): IgA was predominant (2‐3+) with minor co‐reactants including IgM (Cases 1, 2, and 4), C3 (Cases 1 and 3), fibrinogen (Case 3), C1q (Case 4), and light chains (Cases 2, 3, and 4). These trace co‐deposits likely reflect nonspecific mesangial protein trapping, a well‐recognized phenomenon in proteinuric states, and do not independently indicate a proliferative or immune‐complex–mediated process. Fibrinogen, a common nonspecific mesangial reactant in nephrotic syndrome, was present in Case 3 and should be interpreted cautiously.
3. IgA–IgG co‐dominant (Case 5): This pattern warrants particular comment, as IgG co‐dominance raises the differential of IgA‐dominant lupus nephritis or a rare IgA immune‐complex deposition disorder. In our patient, ANA negativity and the absence of systemic features of lupus effectively excluded this diagnosis. Nevertheless, cases with IgA–IgG co‐dominance require systematic exclusion of secondary causes before the diagnosis of MCD with IgA deposits can be accepted.

The mesangial distribution of IgA in all our cases—characteristically forming a granular “garland” or “starburst” pattern along the mesangial stalk, clearly distinct from the subepithelial or subendothelial patterns of membranous nephropathy or lupus nephritis—is a consistent and diagnostically reliable IF feature. The intensity ranged from 1+ to 3+, consistent with published series. Critically, the absence of significant capillary wall IgA staining in our cases further distinguishes this entity from the mesangial‐plus‐capillary‐wall pattern (“mesangial and segmental capillary loop”) seen in more advanced IgAN.

EM was not available at our institution during the study period, representing a significant limitation.

Three main pathogenetic hypotheses explain MCD with IgA deposits. The incidental IgA trapping hypothesis [7] proposes that mesangial IgA deposits represent nonspecific clearance in hyperproteinuria with no pathogenic role, a view supported by similar clinical behavior to classical MCD [2]; however, this fails to explain why IgA should be present when immunofluorescence is classically negative in typical MCD. The dual glomerulopathy hypothesis [1] suggests simultaneous occurrence of subclinical IgAN and T‐cell–mediated MCD. Strong support comes from Cho et al. [3], who demonstrated galactose‐deficient IgA1 (Gd‐IgA1) positivity in all IgAN–MCD patients, indicating pathogenic IgA deposits, and from Rambausek et al. [5], showing T‐cell dysregulation in both conditions. The unified pathogenesis hypothesis proposes that shared T‐lymphocyte dysregulation drives both podocyte injury and mucosal IgA1 overproduction in genetically susceptible individuals, with environmental triggers (upper respiratory and gastrointestinal infections [9], other infections, or toxins [10, 11]) potentially inducing this dual phenotype via immune dysregulation. The diagnostic workup of MCD with IgA deposits must systematically exclude the following conditions: IgAN with nephrotic syndrome. In classical IgAN, nephrotic‐range proteinuria is invariably associated with significant proliferative or sclerosing LM changes (MEST‐C M1, E1, S1, T1–2, and/or C1–2). The diagnosis of MCD with IgA deposits requires the virtual absence of such changes; any Oxford E, S, T, or C lesion favors classical IgAN with superimposed nephrotic syndrome and warrants a different therapeutic approach.

Secondary IgA deposits: Liver cirrhosis, celiac disease, dermatitis herpetiformis, inflammatory bowel disease, and certain infections may be associated with secondary mesangial IgA deposition. A thorough clinical history, serological workup (antiendomysial antibodies and viral serologies), and liver function assessment are essential.

Lupus nephritis with IgA dominance: Rare, but ANA, anti‐dsDNA, and complement consumption are typically present in lupus. Case 5 in our series (IgA–IgG co‐dominance) required this exclusion.

IgA‐dominant postinfectious glomerulonephritis: An acute presentation with elevated ASLO or recent infection history, endocapillary proliferation, and “garland” subepithelial deposits by EM can usually be distinguished clinically and histologically.

Available data suggest that patients with MCD with IgA deposits respond to corticosteroids similarly to classical MCD, with complete remission rates of 60%–80%, though with a tendency toward relapsing‐dependent or relapsing‐remitting courses [2, 3]. In our series, two patients (Cases 1 and 2) experienced multiple relapses necessitating immunosuppressive escalation (cyclophosphamide in Case 1, and ongoing prednisolone in Case 2). The observation that spontaneous remission has been reported in MCD with IgA deposits [3, 4]—unlike typical IgAN—further supports a predominantly MCD‐like behavior in many patients.

Importantly, the presence of IgA deposits does not appear to independently worsen renal prognosis compared to classical MCD in most series [2, 3]. The Oxford MEST‐C score, applicable to the IgAN component of the dual glomerulopathy, may help stratify the risk of progressive renal insufficiency: Patients with exclusively M0‐E0‐S0‐T0‐C0 features are at low risk, whereas those with M1 or S1 lesions may warrant closer surveillance and perhaps RAS blockade in addition to corticosteroids.

## 5. Conclusions

Our series highlights several important points: The diagnosis rests fundamentally on immunofluorescence, with mesangial IgA dominance or exclusivity being the defining criterion; light microscopy may show entirely normal glomeruli or only focal segmental mild mesangial changes, clearly distinct from the proliferative or sclerosing features of classical IgAN; hematuria may herald an underlying IgA‐mediated mesangial process even in patients who subsequently present with a MCD phenotype; the clinical behavior, including steroid responsiveness and relapsing course, more closely resembles MCD than IgAN; and the pathogenesis likely involves overlapping immune dysregulation, possibly with a dual glomerulopathy mechanism in genetically predisposed individuals. Future prospective multicentric studies incorporating EM, Gd‐IgA1 biomarkers, and long‐term renal function surveillance are urgently needed to establish a robust nosological classification and evidence‐based treatment guidelines for this underrecognized syndrome.

## Funding

This research received no external funding.

## Conflicts of Interest

The authors declare no conflicts of interest.

## Data Availability

The data that support the findings of this study are available on request from the corresponding author. The data are not publicly available due to privacy or ethical restrictions.

## References

[1] Bertani T. , Mecca G. , Sacchi G. , and Remuzzi G. , Concomitant Presentation of Three Different Glomerular Diseases in the Same Patient, Nephron. (1983) 35, no. 1, 65–67.

[2] Clive D. M. , Mesangial Immunoglobulin A Deposits in Minimal Change Nephrotic Syndrome: A Report of an Older Patient and Review of the Literature, American Journal of Nephrology. (1990) 10, no. 5, 418–423. 10.1159/000168050 2111636

[3] Cho W. H. , Kim Y. H. , Woo J. S. et al., Characterization of IgA Deposition in the Kidney of Patients With IgA Nephropathy and Minimal Change Disease, Journal of Clinical Medicine. (2020) 9, no. 7. 10.3390/jcm9082619 PMC7464421 32806730

[4] Wu M. J. , Yang Y. K. , Chen Y. M. , and Hsieh Y. D. , Spontaneous Remission of Nephrotic Syndrome in IgA Glomerular Disease, American Journal of Kidney Diseases. (1985) 6, no. 2, 107–111. 10.1016/s0272-6386(85)80148-7 4025333

[5] Rambausek M. , van den Wall Bake A. W. , Schumacher-Ach R. et al., Mesangial IgA Nephropathy and Idiopathic Nephrotic Syndrome, Nephron. (1987) 47, no. Suppl 1, S14–S17. 10.1159/000184489 3683687

[6] Cattran D. C. , Coppo R. , Cook H. T. et al., The Oxford Classification of IgA Nephropathy: Rationale, Clinicopathological Correlations, and Classification, Kidney International. (2009) 76, no. 5, 534–545, 10.1038/ki.2009.243. 19571791

[7] Habib R. , Girardin E. , Gagnadoux M. F. , Hinglais N. , Levy M. , and Broyer M. , Immunopathological Findings in Idiopathic Nephrosis: Clinical Significance of Glomerular Immune Deposits, Pediatric Nephrology. (1988) 2, no. 3, 402–408, 10.1007/bf00853431. 3153051

[8] Choi I. J. , Jeong H. J. , Lee H. Y. , Kim P. K. , Lee J. S. , and Han D. S. , Significance of Mesangial IgA Deposition in Minimal Change Nephrotic Syndrome: A Study of 60 Cases, Yonsei Medical Journal. (1990) 31, no. 2, 97–104, 10.3349/ymj.1990.31.3.258. 2281685

[9] Srinivas S. , Madhavaram S. K. , Bhanushali S. A. , and Kalra K. , The Immunoglobulin A Nephropathy Renaissance: From Pathogenesis to Personalized Therapy, Cureus. (2025) 17, no. 11, 10.7759/cureus.97999. PMC12744310 41466967

[10] Turgutalp K. , Ozhan O. , Gürbüz M. et al., Reversible Minimal Change Nephrotic Syndrome and Glomerular IgA Deposition Associated With Non-Parenteral Heroin Abuse: A Case Report, Medical Principles and Practice. (2012) 21, no. 3, 285–288. 10.1159/000337941 22539034

[11] Boix E. , Lloveras J. , Mariñosa M. et al., Steroid-Responsive Nephrotic Syndrome With Minimal Change and Mesangial IgA Deposits in a HIV-Infected Patient, Nephrology Dialysis Transplantation. (2000) 15, no. 7, 1097–1098. 10.1093/ndt/15.3.412 10692530

